# Shiftwork in the Norwegian petroleum industry: overcoming difficulties with family and social life – a cross sectional study

**DOI:** 10.1186/1745-6673-4-22

**Published:** 2009-08-03

**Authors:** Cathrine Haugene Ljoså, Bjørn Lau

**Affiliations:** 1 National Institute of Occupational Health, PB 8149 Dep, N-0033 Oslo, Norway

## Abstract

**Background:**

Continuous shift schedules are required in the petroleum industry because of its dependency on uninterrupted production. Although shiftwork affects health, less is known about its effects on social and domestic life.

**Methods:**

Consequently, we studied these relationships in a sample of 1697 (response rate 55.9%) petroleum workers who worked onshore and offshore for a Norwegian oil and gas company. We also examined the roles of coping strategies and locus of control for handling self-reported problems with social and domestic life. A questionnaire containing scales from the Standard Shiftwork Index and Shiftwork Locus of Control was answered electronically.

**Results:**

In general, only a few participants reported that their shift schedule affected their social and domestic/family life, and several participants had enough time to spend by themselves and with their partner, close family, friends, and children. Despite this general positive trend, differences were found for shift type and individual factors such as locus of control and coping strategies. Internal locus of control was associated positively with all the dependent variables. However, engaging problem-focused coping strategies were associated only slightly with the dependent variables, while disengaging emotion-focused coping strategies were negatively associated with the dependent variables.

**Conclusion:**

Since most participants reported few problems with social and domestic/family life, the availability of more leisure time may be a positive feature of shiftwork in the Norwegian petroleum industry. Locus of control and the use of coping strategies were important for shiftworkers' social and domestic/family life.

## Background

The petroleum industry is an around-the-clock operation that requires continuous shift schedules. Although some health effects are known to result from such working arrangements, research on how work might interfere with family and social life is insufficient for this industry. Consequently, this study examined the extent to which different shift arrangements in the petroleum industry interfere with family and social life. We also studied how coping strategies and internal locus of control influence the interference that shiftwork has on family and social life.

Based on reports from the petroleum- and other industries, we know that working a nonstandard pattern of days and working hours is associated with conflict between work and family/social life [1-4]; a conflict that might even increase the likelihood of divorce[5,6]. Norwegian offshore petroleum workers spend two weeks offshore followed by a four-week period of shore leave. At onshore installations, most employees follow a continuous six-week shift schedule; five weeks at work followed by one week off.

Problems with family- and social life are experienced in different ways among onshore and offshore petroleum workers. Offshore, life is adapted to activity 24 hours a day. Food and leisure activities are available around the clock, and there are good opportunities for sleep day and night. When the shiftworkers return to home, they are completely free and the families are not exposed to the daily disadvantages of shiftwork. In contrast, onshore workers return to home after each shift, giving rise to family conflicts and disruption of sleep. Separation from the family may be a source of stress for the workers offshore. They must cope with repeatedly parting and reunions, a disrupted family- and social life and other problems relate to the "intermittent husband syndrome"[3,4].

Family conflicts may also influence how well shiftwork is tolerated. A cross-sectional study of nurses engaged in shiftwork found that the partner's experience of shiftwork was a more important factor, when determining whether or not the worker tolerated shiftwork, than were biological factors [7]. A Canadian study found that work-family conflicts mediates the association between shiftwork and depression[8].

Shiftwork is a double-edged sword, on one hand it gives the workers time off to spend with family and friends, on the other hand shiftworkers have to work at unfavorable times of the day[6]. Afternoon shifts may be disruptive because social and family activities take place at this time. Supper, interacting with children and partner, and visiting relatives and friends occur in the early evening [9]; [10]. Consequently, night shifts may be preferred over afternoon shifts because they allow the worker to spend time with family during the early morning and evening hours [9]. Their role as a caregiver may also be a problem for many shiftworkers. Schools and daycare centers are day-shift oriented, which means that the shiftworker and their children may have different schedules and not be able to see each other for days. This may have negative consequences for the children's homework and activities outside the school [11]. An irregular work schedule might also interfere with social activities in everyday life. Cultural, religious and sporting events are often arranged at the weekends, and these may be disrupted by working on Saturdays and Sundays [12]. However, shiftwork might also have its advantages. Some shiftworkers have reported that they chose shiftwork to improve their families' childcare arrangements [13].

Internal locus of control is an important consideration for overcoming the effects of shiftwork. Locus of control has its roots in Social Learning Theory; in particular, Rotter's notion that expectancy is 'the probability held by the individual that a particular reinforcement will occur as a function of a specific behavior on his part in a specific situation or situations' [14]. In other studies of shiftworkers, internal locus of control has predicted better sleep, less fatigue, better health, better adjustment to shift rotation, and less disruption to life outside of work [15-19]. It has been assumed that there are two reasons why individuals with a high shift-work-specific internal locus of control suffer fewer shift-related problems. First, such people would initiate more self-regulatory efforts at preventing or controlling problems related to shiftworking because of their belief that they are responsible for the outcomes they experience. Second, to do so, such people would search for information on how to control the shift-related difficulties they have experienced.

Individual coping strategies are also important for examining how shiftwork influences a worker's social and family life. The literature differentiates between coping on one side and mastery and self-efficacy on the other. In the literature the term "coping" is referred to as the process of reacting to external demands. That is, what one think and do, and the coping strategies being used. Mastery is experienced when these responses lead to the desired result. Self-efficacy is the expectation about the ability to solve the problem or assignment [20]. Several experimental studies have showed that neuroendocrine activity normalises, and that progress of illness is prevented if one experience control over the situation and self-efficacy[21]. Research indicates that lack of control in combination with difficult tasks, can result in health problems. This implies that action aimed at coping with shift work can be directed at the exposure, or at actions that can give the shiftworker new possibility to cope, while the registered outcome is the experience of mastering the challenges of shift work.

In a study of shiftworking nurses, socializing strategies, such as participating in sports and hobbies and keeping in contact with other shiftworkers, were positively associated with better social life, better psychological well-being, fewer sleep problems and higher job satisfaction [22]. In a study with nurses, Costa [23] found that the use of an emotional expressive coping strategy increased the support received from the family. Similarly, Pisarski, Bohle and Callan [24,25] found that ambulance service workers who received support from their families were less likely to use an emotional avoidance coping strategy.

However, there is also the possibility that workers who have coped well with shiftwork do not need to use a lot of active coping strategies. A qualitative study among nurses showed that those with physical or personal qualities that did not fit them for night work, depended to a greater extent on active coping strategies to manage night work [26]. Consequently, it could be that shiftworkers who are able to sustain a good social or family life do not need to use active coping strategies.

The research reported, which so far has been conducted mainly in the healthcare and service industries, cannot be generalized to relate to the petroleum industry. To our knowledge, there have been no studies in the literature referring to how shiftwork might interfere with family and social life, and how different coping strategies and locus of control might influence such relationships, among employees in the petroleum industry. Because of this lack of knowledge, we examined, first, how different shift arrangements in a large Norwegian petroleum company might interfere with family and social life. Second, we examined how coping strategies and locus of control influence these relationships when shift arrangement has been controlled. More specifically, we expected that a high shiftwork-related internal locus of control and engaging coping strategies would be associated with lower levels of reported problems with social and domestic life, irrespective of the type of work schedule. In addition, we expected that locus of control would be associated with a greater use of engaging problem-focused coping strategies, such as seeking social support and coping by problem solving.

## Method

### Procedure

All 3038 employees of a large Norwegian oil and gas company, who worked onshore and offshore during a two-week period in August 2006, were invited to participate in the study (see Table 1 for further details). The research design used a web-based questionnaire. Researchers at the National Institute of Occupational Health received a list of all the employees. To generate an ID number and *"Subject Access Codes" *for the web-based questionnaire, the list contained each participant's name, gender, age, organizational belonging, and occupational title. All employees were sent a personal written invitation to participate in the research project through the internal mail at their workplace. The invitation consisted of general information regarding the purpose of the study and their personal access code for completing the questionnaire. The data were collected within the same two-week period in August 2006. Complete questionnaires were received from 1697 employees, yielding a response rate of 55.9%. As shown in Table 1, slightly more men than women answered the questionnaire.

**Table 1:** Participation in relation to gender, age, marital status, and workplace.

		Invited	Participated		
		Number	Number	Percent	χ^2^
					
Total		3038	1697	55.9	
					
Gender	Women	582	299	51.4	5.87*
	Men	2456	1398	56.9	
					
Age group	20 – 29	282	156	55.3	6.84
	30 – 39	701	403	57.5	
	40 – 49	1027	592	57.6	
	50 – 59	887	478	53.9	
	≥ 60	141	68	48.2	
					
Marital status	Married		1430		§
	Single		202		
					
Workplace	Offshore	2406	1336	55.5	0.52
	Onshore	632	361	57.1	

§No information was received about marital status. * *p *< .05

### Measurements

#### Background variables

Information about gender, age, and occupation was provided by the company. Participants were asked to state their marital status in terms of the following categories: 1) married/partner/cohabitant, 2) separated/divorced, 3) widow/widower, and 4) single. Because of their low response rates, categories 2 to 4 were combined in the analyses, resulting in the two categories: 1) married/cohabitant, and 2) single.

#### Shift arrangements

Offshore petroleum workers spend two weeks offshore followed by a period of shore leave. On Norwegian installations, offshore tours of duty are normally limited to a maximum of two weeks. Currently, the most frequently worked pattern is two weeks offshore, alternating with four weeks shore leave. Specialist personnel, who frequently move between different installations, often have irregular and/or unpredictable work patterns. At any one time, only two crews can be accommodated on board; thus, the standard shift duration is 12 hrs for day/night shiftworkers operating continuous processes such as drilling and production. The shift duration for day workers offshore is also 12 hrs. Therefore, a two-week tour of duty involves a minimum of 168 hrs work, although some personnel (especially managers and supervisors) may work longer hours[27].

At onshore installations, most employees' work is performed on a continuous six-week shift schedule; five weeks at work being followed by one week off. A typical shift-work schedule would be: first week, two morning and three night shifts; second week, three afternoon shifts; third week, four night shifts; fourth week, four morning shifts; fifth week, three morning shifts; and sixth week, time off.

Participants were asked to specify the type of shift rotation they worked. For employees working offshore, the alternatives were: 1) 14 days on, 28 days off (mainly day work); 2) 14 days on, 28 days off (one two-week day period, one two-week night period); 3) 14 days on, 28 days off (mainly night work); 4) 14 days on, 28 days off (first week, day; second week, night); 5) 14 days on, 28 days off (2 work periods day/1 work period night); and 6) other arrangements. As few employees working offshore chose categories 3 to 6, these categories were combined into one category. For employees working onshore, the alternatives were: 1) a continuous six-shift schedule; 2) a 24-hour shift; and 3) other arrangements. Because only a few onshore workers chose categories 2 and 3, these categories were combined with category 1. Consequently, the following work-time arrangements were used in the analyses: 1) Offshore, day work (656 persons); 2) offshore, one day period/one night period (474 persons); 3) other arrangements offshore (206 persons); and 4) onshore (361 persons).

### Social and Domestic Survey

Five questions were taken from the Social and Domestic Survey included in the Standard Shiftwork Index Survey [28]. These questions measure the extent to which participants felt that their shift schedule gave them enough time to spend by themselves and with their family, friends and children. Using five-point Likert scales, where 1 indicated "not at all", 3 indicated "somewhat" and 5 indicated "very much", the following mean scores and standard deviations (SD) were obtained for the questions "Are you satisfied with the amount of time your shift system leaves you for: 1) your partner (mean: 4.03; SD: 0.81); 2) your close family (mean: 3.71; SD: 0.89); 3) friends and social relations (mean: 3.35; SD: 0.94); 4) your children (mean: 3.98; SD: 0.86); and 5) yourself (mean: 3.84; SD: 0.93)".

### Global questions

Two questions that addressed the effect of shiftwork on social and domestic life were taken from the Standard Shiftwork Index [28]. Using five possible response categories (1 indicated "never", 3 indicated "somewhat", and 5 indicated "always"), the respondents were asked: "In general, to what extent does working shifts cause you problems with social life (mean: 2.67; SD: 0.87) and domestic life (mean: 2.55; SD: 0.87)".

### Coping style

The Coping with Shiftwork Questionnaire (CSQ) [29] is a scale developed for use in the shiftwork context and included in the Standard Shiftwork Index [28]. Since this questionnaire had not been used previously in Norway, it was translated into Norwegian. The CSQ measures eight different strategies that may be used to cope with challenges associated with shiftwork in four areas. So that not too many questions were included in the questionnaire, questions relating to five coping strategies (problem solving, cognitive restructuring, social support, wishful thinking and self-criticism) in three areas (social life, family and domestic life, and job performance) were included in this study. This yielded a total of 15 questions (three questions covering each coping strategy in five areas). Each question had five answer categories: 1) not used, 2) used a little, 3) used somewhat, 4) used quite a bit, and 5) used a great deal. A principal components factor analysis with a varimax rotation supported a five-factor solution with loadings for the five coping strategies. As a result, five mean scores were computed, based on the three questions that loaded on each of these five dimensions. The resulting five coping strategies can be further classified according to approach (engagement versus disengagement) and focus (problem-focused versus emotion-focused). Consequently, in this study, we used two problem-focused engaging coping strategies; problem solving (cronbachs alpha; .84) and seeking social support (cronbachs alpha; .85), one emotion-focused engaging coping strategy; cognitive restructuring (cronbachs alpha; .83), and two emotion-focused disengaging coping strategies; wishful thinking (cronbachs alpha; .90) and self-criticism (cronbachs alpha; .89). Mean scores on these scales were: problem solving, mean = 2.91, SD = 0.89; support seeking, mean = 2.66, SD = 0.90; cognitive restructuring, mean = 3.23, SD = 0.86; wishful thinking, mean = 2.12, SD = 0.95; and self criticism, mean = 2.14, SD = 0.87.

### Shiftwork Locus of Control

Rotter [30] introduced a scale that measured a general locus of control, which was followed later by the development of several more domain-specific instruments. Smith, Spelten and Norman (1995) introduced the Shiftwork Locus of Control (SHLOC) scale, which measures locus of control in relation to shiftwork. The SHLOC scale is an internally oriented measure that allows respondents to be placed on a continuum from low to high shiftwork-specific internal locus of control. The original version of the scale contains 20 items that measure internal beliefs relating to the four shiftwork-related areas: sleep, social life, health, and work (five items each). The instrument satisfies the requirements regarding reliability and validity [31]. Because the SHLOC scale had not been used previously in Norway, it was translated into Norwegian. Concerns about the length of the questionnaire resulted in a shortened version of the SHLOC scale being used in this study. Only two questions were used from each of the four dimensions in the original English version of the instrument. A principal components factor analysis with varimax rotation supported a four-factor solution, as expected. In the following analyses, we used the following two questions from the Social dimension: 1) "When working shifts I determine whether or not I have a proper social life"; and 2) "When I work shifts it is my own fault if my social life suffers". These questions had six response categories: 1) strongly disagree, 2) somewhat disagree, 3) slightly disagree, 4) slightly agree, 5) somewhat agree, and 6) totally agree. Cronbach's alpha for this scale was .85, and the mean score was 3.37 (SD = 1.28).

### Statistical analysis

GLM univariate analyses of variance and post hoc Bonferroni tests were conducted to find differences between the various shift schedules on questions covering family and social life. These analyses controlled for gender, age, and civil status. Further, multiple regression analyses were carried out to determine whether shiftwork locus of control and coping strategies had any effect on family and social life. Multiple regression analyses where also carried out to determine if interactions between location (onshore vs offshore) and individual differences (shiftwork locus of control and coping strategies) were able to predict the social and domestic outcome variables, controlled for main effects. Pearson correlations were used to test the hypothesis that shiftwork locus of control is associated with engaging problem-focused coping strategies, such as seeking social support and problem-solving coping. SPSS version 17 was used to perform these analyses.

### Ethics approval

The research data were anonymous as all names and personal ID numbers were omitted. The study was conducted in accordance with the World Medical Association Declaration of Helsinki and with permission from the Data Inspectorate of Norway.

## Results

As indicated by the estimated mean scores in Table 2, we did not find especially high scores on questions that examined whether shiftwork produced problems with social life and family life. At the same time, the general finding was that the participants experienced ample time to spend by themselves and with their partner, family, friends, social relations, and children. However, employees in the various shift systems experienced this differently. The highest mean scores on those questions that addressed whether shiftwork caused problems with social life and domestic/family life were found among employees working "one day period and one night period offshore" and those with onshore shift arrangements. These two groups could be distinguished from the "day work offshore" and "other arrangements offshore" groups. Compared with employees who worked offshore, the onshore employees also scored less favorably on all questions concerning time spent by themselves and with their partner, family, friends, social relations, and children.

**Table 2:** Estimated mean values of relations involving social/family life, adjusted for age, gender, and civil status.

		Shiftwork gives problems with social life	Shiftwork gives problems with domestic/family life	Enough time with partner	Enough time with close family	Enough time with friends and social relations	Enough time with your children	Enough time for yourself
Shift schedule	Offshore, day work	2.38^ab^	2.27^ab^	4.15^ab^	4.03^ab^	3.77^ab^	4.24^a^	4.21^ab^
	Offshore, one day period, one night period	2.75^ac^	2.62^ac^	4.00^ac^	3.78^acd^	3.51^acd^	4.11^bc^	3.97^acd^
	Other arrangements offshore	2.40^cd^	2.34^c^	4.18^d^	4.05^ce^	3.84^ce^	4.32^bd^	4.25^ce^
	Onshore	2.71^bd^	2.54^b^	3.63^bcd^	3.44^bde^	3.15^bde^	3.77^acd^	3.77^bde^

Note: Values with similar letters are significantly different at the .05 level (Bonferroni test). High values indicate that the respondent agrees with the relation being measured.

Multiple regression analyses (Table 3) showed that, when working shifts, internal locus of control with respect to one's social situation was associated negatively with problems in social and domestic/family life, and positively with having enough time for partner, family, friends, children, and oneself. Use of problem solving as a coping strategy was associated with problems with domestic/family life. Social support as a coping strategy was associated with having enough time to be with friends and to maintain adequate social relations. Further, wishful thinking as a coping strategy was associated with problems in social and domestic/family life. High scores on self-criticism were associated with all of the dependent variables. Self-criticism as a coping strategy was associated with more problems in the worker's social and domestic/family life and with having less time for partner, family, friends, children, and oneself.

**Table 3:** Multiple regression analyses for problems with social/domestic life, and the experience of having enough time.

	**Dependent variables**						
	Shiftwork gives problems with social life	Shiftwork gives problems with domestic/family life	Enough time with partner	Enough time with close family	Enough time with friends and social relations	Enough time with your children	Enough time for yourself
Predictors							
							
Locus of control social life	-0.39**	-0.36**	0.25**	0.29**	0.25**	0.23**	0.19**
Problem solving as coping	0.04	0.10*	-0.09	-0.08	-0.05	-0.06	-0.08
Social support as coping	-0.02	-0.02	0.07	0.06	0.08*	0.07	0.05
Cognitive restructuring	0.02	-0.03	0.02	-0.01	-0.02	0.03	0.04
Wishful thinking as coping	0.10*	0.12**	-0.03	0.02	-0.05	-0.04	-0.04
Self-criticism as coping	0.19**	0.16**	-0.10*	-0.13**	-0.12**	-0.12*	-0.16**

* p < .01, ** p < .001

Note: All predictors were entered simultaneously and were adjusted for gender, age, marital status, and shift schedule. Beta values are reported.

A regression analysis was conducted to see if there were any significant interactions between onshore/offshore location and individual differences like shiftwork locus of control and individual coping strategies in predicting the outcome variables. No such interactions were found.

As shown in Table 4, the hypothesis that shiftwork locus of control would be associated with engaging problem-focused coping strategies, such as seeking social support, and problem-solving coping was not supported. However, locus of control was negatively associated with both types of emotion-focused disengaging coping strategies (wishful thinking and self-criticism).

**Table 4:** Correlation matrix showing Pearson correlations between locus of control on social life for all coping strategies.

	1	2	3	4	5	6
1. Locus of control social life	1					
2. Problem solving as coping	-.04	1				
3. Social support as coping	-.02	.57*	1			
4. Cognitive restructuring as coping	.03	.62*	.50*	1		
5. Wishful thinking as coping	-.19*	.25*	.28*	.14*	1	
6. Self-criticism as coping	-.10*	.31*	.27*	.17*	.59*	1

* Correlation is significant at the .001 level (2-tailed).

## Discussion

In general, low scores were obtained for questions that sought information on whether the shift schedule caused problems with social and domestic/family life. High values were given for questions that sought information on whether shiftworkers had enough time to spend by themselves and with their partner, close family, friends, and children. This may reflect the positive side of shiftworking in the Norwegian petroleum industry, that is, more leisure time. Despite this general positive trend, differences were found according to shift type and individual factors such as coping strategies and internal locus of control.

Employees working onshore had higher scores on those outcome variables that indicated whether shiftwork was responsible for problems with social and domestic life, respectively. These employees also had low scores on all the questions related to having enough time to spend by themselves and with their partner, close family, friends, social relations, and children. As mentioned in the introduction, the problem for the onshore workers may be that they live at home in their normal environment where, to some extent, they have to adjust to the demands from family, friends, and the rest of the community [9-12]. Perhaps these challenges do not occur for offshore workers, who are separated from the rest of society for two-week periods. However, we should not ignore the possibility that the differences found between onshore and offshore employees may not necessarily imply that onshore employees are worse off than normal workers on these variables. The reason may be that the four-weeks' leave available to employees who work offshore provides enough time for themselves and for their pursuit of a social life.

Among offshore workers, the shift arrangements that seemed to be associated with the most social and domestic/family problems were "one day period and one night period offshore." These problems may be caused by night work. Working at night requires employees to change their daily rhythm after 14 days with a 12-hour night shift in order to adapt to a normal daily rhythm. Consequently, they require several days before adapting to the daytime rhythm of life with their family and friends.

In general, engaging problem-focused coping strategies, which are considered best for coping in situations that can be influenced by one's experience, were not associated with the outcome variables in this study. However, there were two exceptions to this general finding. The *problem-solving *coping strategy was significantly and positively associated with problems in domestic/family life. This may result from a correct use of coping strategies; those who have problems with domestic/family life use more coping strategies than those who experience fewer problems. The second exception was that, perhaps not surprisingly, *seeking social support *was positively associated with having enough time to be with friends and to maintain social relations. This is consistent with the findings in Henderson et.al[22] study. The lack of further correlations between these engaging problem-focused strategies and the outcome variables may have resulted from the low scores on questions that referred to the lack of time employees had for social relations, and the resulting self-reported problems. If these conditions were not experienced as problematic, we would not expect to find differences between employees who used few or many of these engaging and problem-solving coping strategies. However, the lack of significant correlations could also have resulted from the employees not having the opportunity to influence the outcome variables.

A different pattern of findings emerged for the disengagement and emotion-focused coping strategies, such as self-criticism and wishful thinking. Self-criticism was associated with all of the dependent variables and wishful thinking was associated with self-reported problems in both social and domestic/family life. However, we cannot determine whether there is a causal relationship between these coping strategies and the outcome variables, as we only used cross-sectional data. It may be that the use of these coping strategies causes problems in both social and family life, but it is also possible that those who have problems in these areas employ these coping strategies. A third possibility is that another factor, such as personality, causes some of the employees to score high on both outcome variables and these coping strategies. However, in general, the use of emotion-focused disengaging coping strategies will not result in any change in the situation. Fortunately, none of these coping strategies was used to any great extent by our sample of employees.

Although cognitive restructuring was the most frequently reported coping strategy in this study, it was not associated with any of the dependent variables. This may result from it not being a very effective strategy. Cognitive restructuring is an engaging emotion-focused coping strategy, the effect of which might be that shiftworkers can focus on positive aspects of their shift schedule to compensate for any negative effects. Could it be that the high incidence of this form of compensation indicates that participants focused on positive aspects of their shiftwork arrangements, such as the long rest periods, but that this, in itself, does not solve the problems they might experience with their family and social life?

Support was obtained for the hypothesis that shiftwork-specific internal locus of control is negatively associated with reported problems with social and family life, and is positively associated with having enough time to spend in these areas, irrespective of the type of work schedule. This finding accords with the locus of control literature, which reports benefits of an internal orientation among shiftworkers when dealing with shift-related problems [19]. The hypothesis that locus of control is associated with engaging problem-focused coping strategies (e.g., seeking social support and problem solving) was not supported. As stated earlier, internal locus of control is a form of perceived control that reflects generalized expectancies about reinforcement contingencies. It is important, however, to distinguish between perceived control and the actual execution of control [32]. In theory, locus of control would primarily predict behavior in stressful situations. When confronted by stressors, internals tend to react in a more constructive fashion than do externals, such as actively looking for solutions [33,34]. In other words, the lack of associations between locus of control and engaging-coping strategies might reflect the fact that the outcome variables used in this study were not particularly problematic for the respondents. The execution of an actual behavior also depends on the value placed on a given outcome. Although such a 'value' measure was not incorporated in this study, it might have had a moderating effect on the relationship between locus of control and engaging coping strategies, since positive associations between locus of control and active coping strategies might depend on such a value being placed on a particular outcome.

Nevertheless, locus of control was associated with fewer self-reported problems and more reported time with family and social relations, and by oneself. Consequently, we cannot exclude the possibility that internals deviate from the coping strategies measured in this study in a manner that is beneficial for undertaking these challenges. Therefore, it would be important to identify in more detail what this group actually does to handle their social life when working shifts. Another possible explanation for the associations between locus of control and the outcome variables is that the perception of control of social and family life might ameliorate any potentially negative impact in these areas. It is fairly well established that, whether or not actual control is available and can be executed, the belief that personal control is possible can moderate the outcome.

The fact that so few of the participants reported having internal locus of control of their own social lives is worrying. Locus of control is generally considered to refer to a relatively stable set of beliefs about the relationships between events and the locus of causality of these outcomes. However, Lefcourt [35] rejected the idea of locus of control as a rigid personality characteristic and argued that it was amenable to change in response to life experiences, such as the acquisition of social and work-related skills. In an examination of work locus of control, Daniel and Guppy [36] offered some support for the changeability hypothesis, when, as a result of test-retest data, they concluded that work locus of control could be considered to be more a state than a trait variable. Consequently, the low levels of locus of control in this sample may reflect the actual situation in which the employees do not have control over conditions that influence the association between their shiftwork and their social and family situations. It is important to determine if this is the case, and if so, to look for obstacles for taking control and responsibility over their own social life when they work shifts. It is also worth considering whether any perceived lack of control might be the long-term result, rather than the cause, of chronic fatigue, sleep disturbance, performance shortfall or ineffective coping.

### Strengths and weaknesses

The somewhat low response rate may be a result of various factors. First, the participants are used to the company's own computer system, and may have found it difficult using an unknown system. Secondly, the organization may suffer from tiredness because of answering questionnaires. A third factor may be related to sick leave. There will always be some employees who are absent due to sickness and therefore cannot answer the questionnaire.

Despite the low response rate, the sample was unbiased, except for gender. There were more dropouts among women than men. Although the sample is probably representative, despite the somewhat low response rate, it may contain another source of error: the so-called "healthy worker effect". This means that people working shifts are already a selected group of people [37]. Also, there is always a risk that personal or contextual factors may influence whether an individual responds to a survey or not, which in turn may produce biases of relevance to this study.

The survey was cross-sectional, implying that the results were only concerned with the situation in August 2006. Because the study was not prospective, we cannot infer anything about causality or development over time. All employees working during the two weeks the survey was open were invited to participate. This is a strength of the study as the results can be generalized; its large sample size also strengthening the assumption that the sample is representative.

One could argue that the study is weakened by the reliance of single-item measures, and that the single-items that comprise the dependent variables could have been used as one (or two) scales. However, this is problematic because of the different number of respondents to these questions. Because not everyone has family and/or children, such a construction of scale (s) would cause many of the participants not to be included in the analyses.

Another concern was that the dependent variables used in Table 2 and Table 3 were inter-correlated. Consequently, an initial MANOVA was carried out to determine which predictors influenced the set of dependent variables as a whole. In this analysis, which is not shown in this article, all the dependent variables were entered simultaneously, and gender, age, shift-type, marital status, shiftwork locus of control and all the coping strategies scales were entered as independent variables. In general, the results of this analysis allowed us to use the dependent variables in separate analyses, as shown in Table 2 and Table 3, respectively.

## Conclusion

The majority of the shiftworkers in this study reported few problems with social and domestic/family life, and they had more than enough time to spend by themselves and with their partner, close family, friends, social relations, and children. However, employees in the various shift systems experienced different effects of shiftwork. The highest mean scores on questions related to problems shiftwork might impose upon an employee's social and domestic/family life were found among those working "one day period and one night period offshore" and those with onshore shift arrangements. Locus of control and the use of coping strategies were important for how shiftworkers experienced their social and domestic/family life when working shifts. Internal locus of control was associated negatively with problems in the employee's social and domestic/family life, and positively with the employees having enough time to spend by themselves and with their partner, family, friends, and children. The hypothesis that shiftwork locus of control is associated with engaging problem-focused coping strategies (seeking social support and problem solving) was not supported. However, locus of control was negatively associated with emotion-focused disengaging coping strategies (wishful thinking and self-criticism).

The results might have implications on an individual level. Locus of control and the use of emotion-focused disengaging coping strategies are associated with problems regarding family- and social life. On an organizational level, this may imply that experience of having control is important for shiftworkers. On the other hand, this is a cross-sectional study, so we cannot infer anything about causality.

## Competing interests

The authors declare that they have no competing interests.

## Authors' contributions

Both authors were involved in conception and design, acquisition, analysis and interpretation of data and writing of the manuscript.
